# Clinical course of spinal pain in adolescents: a feasibility study in a chiropractic setting

**DOI:** 10.1136/bmjopen-2024-088834

**Published:** 2025-01-30

**Authors:** Laura RC Montgomery, Steven J Kamper, Anika Young, Amber Beynon, Katherine A Pohlman, Lise Hestbæk, Mark J Hancock, Simon D French, Christopher G Maher, Michael S Swain

**Affiliations:** 1 Faculty of Medicine and Health, The University of Sydney, Sydney, New South Wales, Australia; 2 Department of Chiropractic, Faculty of Medicine Health and Human Sciences, Macquarie University, Sydney, New South Wales, Australia; 3 Curtin School of Allied Health, Faculty of Health Sciences, Curtin University, Perth, Western Australia, Australia; 4 Research Center, Parker University, Dallas, Texas, USA; 5 Department of Sports and Clinical Biomechanics, University of Southern Denmark, Odense, Denmark; 6 Department of Health Sciences, Faculty of Medicine Health and Human Sciences, Macquarie University, Sydney, New South Wales, Australia

**Keywords:** Adolescent, Back pain, Prognosis

## Abstract

**Abstract:**

**Design:**

Prospective feasibility study.

**Objectives:**

To inform the design and conduct of a large-scale clinical cohort study investigating adolescents with moderate-to-severe spinal pain.

**Setting:**

Chiropractic care in Sydney, Australia.

**Participants:**

Adolescents aged 12–17 years with spinal pain (≥4/10 pain intensity score).

**Methods:**

Adolescents and chiropractors completed baseline and week-12 follow-up questionnaires, with adolescents reporting pain intensity and recovery weekly via text messages during weeks 1–11. Questionnaire measures included spinal pain, pain coping, quality of life, physical activity, clinical assessment findings and care delivered. Chiropractors provided usual clinical care. We conducted a descriptive feasibility analysis.

**Primary outcomes:**

(1) Recruitment rate, (2) response rate to each data collection instrument and (3) retention rate.

**Results:**

From May 2021 to February 2023, 20 chiropractors from 10 clinics were enrolled (invited n=85). 10 chiropractors recruited 45 adolescents (15.4±1.4 years, 43% female) over 13.5 months, excluding an 8-month pause due to COVID-19 disruptions. The average recruitment rate was 0.6 adolescents/recruiting chiropractor/month. We achieved a 100% response to chiropractor baseline and follow-up questionnaires, 98% to adolescent baseline, 94% average response to combined weekly text messages and 93% retention of adolescents at study completion.

**Conclusions:**

Our high response and retention rates demonstrate feasible data collection methods in this population. Addressing low recruitment by expanding the number and type of clinicians is necessary for a successful larger study.

**STRENGTHS AND LIMITATIONS OF THIS STUDY**

- This study used a prospective clinical cohort design to investigate the feasibility of studying adolescents with moderate to severe spinal pain in Australian chiropractic care.
- Weekly data collection tracked pain intensity and recovery, providing valuable insight into the clinical course of spinal pain in adolescents.
- As a feasibility study, our primary focus was assessing recruitment, data collection methods and retention. As such, our findings regarding clinical courses are descriptive and should be interpreted with caution.

## Introduction

### Background

 Spinal pain, pain felt in the neck and mid or lower back, is common during adolescence. Approximately one-third of young people experience spinal pain each month, and up to one-quarter experience chronic or recurrent spinal pain lasting longer than 3 months.^1 2^ Ongoing pain significantly impacts adolescents and their families, leading to reduced participation in school, sports, play and social activities and increased use of medication and healthcare services.^3^ Spinal pain can also worsen mental health, including heightened anxiety and fear of future pain^4^ and is linked to higher odds of alcohol and tobacco use.^5^ The burden of spinal pain extends beyond the teenager and their family, significantly affecting society. From age 10 to 20, low back pain progresses from the fifth cause of years lived with disability to the first and is the primary cause of non-fatal health loss globally.^6^

Adolescence is a critical period for establishing health behaviours, which can significantly impact long-term health, including susceptibility to chronic disease later in life.^7^ Moderate-quality evidence suggests that a history of back pain is a risk factor for future episodes.^8^ Specifically, persistent low back pain during adolescence quadruples the odds (OR 4.3, 95% CI 3.5 to 5.3) of persistent low back pain in adulthood.^9^ Unlike in adults, the prognosis, clinical course and response to treatment for adolescents with spinal pain may be influenced by developmental factors, such as biological maturity and psychosocial changes.^10 11^ Accordingly, applying findings from the extensive adult research to younger populations would be problematic, highlighting the need to expand the evidence base on spinal pain in adolescents.^12 13^

Understanding the overall prognosis and prognostic factors for spinal pain in adolescents is crucial for designing effective healthcare approaches, informing clinical practice and shaping healthcare policy. Despite the burden of adolescent spinal pain, little evidence exists identifying individuals at high risk of ongoing pain.^14 15^ Additionally, research investigating the clinical course of spinal pain in adolescents seeking healthcare is lacking.^16 17^ Effective management of spinal pain in young people may yield long-term benefits in preventing and reducing chronic spinal pain later in life.^18^

### Objectives

This study aimed to inform the design and implementation of a large-scale prospective clinical cohort study investigating the clinical course of moderate-to-severe spinal pain in adolescents seeking health care. Our feasibility objectives were to determine the (1) recruitment rate, (2) response rates to each data collection instrument and (3) retention rate at study completion.

## Methods

### Study design and management

We conducted a clinical cohort feasibility study and report adhering to the Consolidated Standards of Reporting Trials (CONSORT) extension for pilot trials supplemented by the Strengthening the Reporting of Observational Studies in Epidemiology (STROBE) checklist for reporting observational studies. Study data were collected and managed using Research Electronic Data Capture (REDCap). No changes were made to the study methods after commencement.

### Setting and study size

Between 12 May 2021 and 12 December 2022, we invited chiropractors in Sydney, Australia, to participate in the study on an ongoing basis until a total of 20 agreed to recruit adolescents. Chiropractors received study protocol and data collection training from study investigators in person or online, according to preference and COVID-19 protocols. Chiropractors were instructed to screen all consecutive adolescents presenting for care to minimise sampling bias. We sent fortnightly emails to remind chiropractors of study protocols and to sustain motivation for participant screening. Recruitment continued until the target sample size of 45 adolescents had been achieved. The sample size was pragmatically determined based on available resources (including time, funding and logistical considerations) and prior study investigator experience. We estimated an 89% retention rate, accounting for five participants expected to drop out, providing sufficient precision within a 95% CI, with the lower bound remaining around 80%.^19 20^

### Adolescent participants and eligibility criteria

Chiropractors invited adolescents who presented for care to participate. Eligibility was determined using an online screening questionnaire (online supplemental resource 1). Adolescents were eligible if they experienced: (1) moderate-to-severe pain intensity, ≥4/10^21^ on the numeric rating scale (NRS) at the time of presentation; (2) pain in the neck and mid or lower back (as indicated on a body diagram), with or without radiating or referred pain; (3) onset within the past 6 weeks, preceded by 4 weeks without pain (0/10 NRS) and (4) no indication of specific pathology (ie, fracture, infection, malignancy, inflammatory or congenital disorder). Additionally, adolescents had to meet criteria for age (12–17 years), language (English) and connectivity (SMS and email). Eligible adolescents and their parents/guardians were provided information about the study and a consent form. Chiropractors were requested to keep records of ineligible adolescents, those who declined participation and reasons for exclusion and non-participation. All adolescents received the usual clinical care from their chiropractor.

### Study processes and variables

Adolescents meeting the eligibility criteria were enrolled in the study from 28 May 2021 to 13 February 2023. Upon assent, adolescents were directed to complete an online questionnaire before their first appointment. This questionnaire captured patients demographics, spinal pain assessed using the Young Spine Questionnaire (YSQ),^22^ recovery expectations, medication usage, quality of life measured by the Paediatric Quality of Life Inventory (PedsQL), Paediatric Pain Coping Inventory (PedsQL-PPCI), Multidimensional Fatigue Scale (PedsQL-MFS) and specific items from the Youth Risk Behaviour Survey to evaluate physical activity (refer to online supplemental resource 1). PedsQL-PPCI scores were transformed to a scale of 0–2, while all other PedsQL measures were scaled from 0 to 100, with higher scores indicating better coping/function.^23^ Within 24 hours of the adolescent’s first appointment, the chiropractor received an online baseline questionnaire via email, capturing the adolescent’s spinal pain history, presentation and physical assessment, including Beighton’s hypermobility scale and fingertip-to-floor distance (online supplemental resource 1).

Over the following 11 weeks, two text messages (SMS) were sent via Twilio for REDCap to each adolescent every Sunday. SMSs contained a web survey link asking adolescents to report their pain course via their average pain intensity on the 11-point NRS and their perceived pain recovery on the 11-point Global Back Recovery Scale (GBRS) (online supplemental resource 1). The NRS ranges from zero (‘no pain’) to 10 (‘worst imaginable pain’). The GBRS ranges from negative five (‘very much worse’) through zero (‘no change’) to positive five (‘completely recovered’).

At week 12, adolescents received an SMS link to a final online follow-up questionnaire, which captured pertinent repeat baseline information, details of chiropractic care delivered, satisfaction level and the occurrence of adverse events (online supplemental resource 1). Chiropractors received an online follow-up questionnaire via email, which captured the clinical care provided (online supplemental resource 1). All instrument completion times were recorded based on online access and submission timestamps. Adolescents were reimbursed AU$20 for their time completing baseline surveys and an additional AU$20 for follow-up questionnaires. Chiropractors were reimbursed AU$80 per adolescent for their time completing study procedures.

### Non-response and incomplete data collection

An automatic electronic reminder was sent 24 hours later via the same contact method (SMS or email) for non-response or incomplete data. If there was still no response, we telephoned the adolescent or their parent/guardian three days later. Loss to follow-up was defined as failure to respond to the reminders and subsequent data collection instruments. Research staff attempted to contact adolescents who were lost to follow-up via telephone for an exit interview to collect their follow-up questionnaire data.

### Statistical methods

We calculated the adolescent recruitment rate, response rate to each data collection instrument and retention rate at study completion. Descriptive statistics characterise the cohort at baseline, compare baseline scores between adolescents completing week 12 follow-up and those lost to follow-up and assess outcome changes between baseline and follow-up. Survival analysis estimated the probability of recovery within the 12-week study period. There is no universally accepted single measure of recovery from spinal pain. We defined *recovery* from the episode of spinal pain at enrolment as two consecutive weeks reporting ‘no pain’ (0/10 NRS), with recovery recorded at the start of this period. Adolescents lost to follow-up were censored at the last point when data were received. Data were analysed using R V.4.3.0.

### Patient and public involvement statement

There was no patient or public involvement in this study’s design, conduct, reporting or dissemination.

## Results

### Feasibility

**Figure 1:**
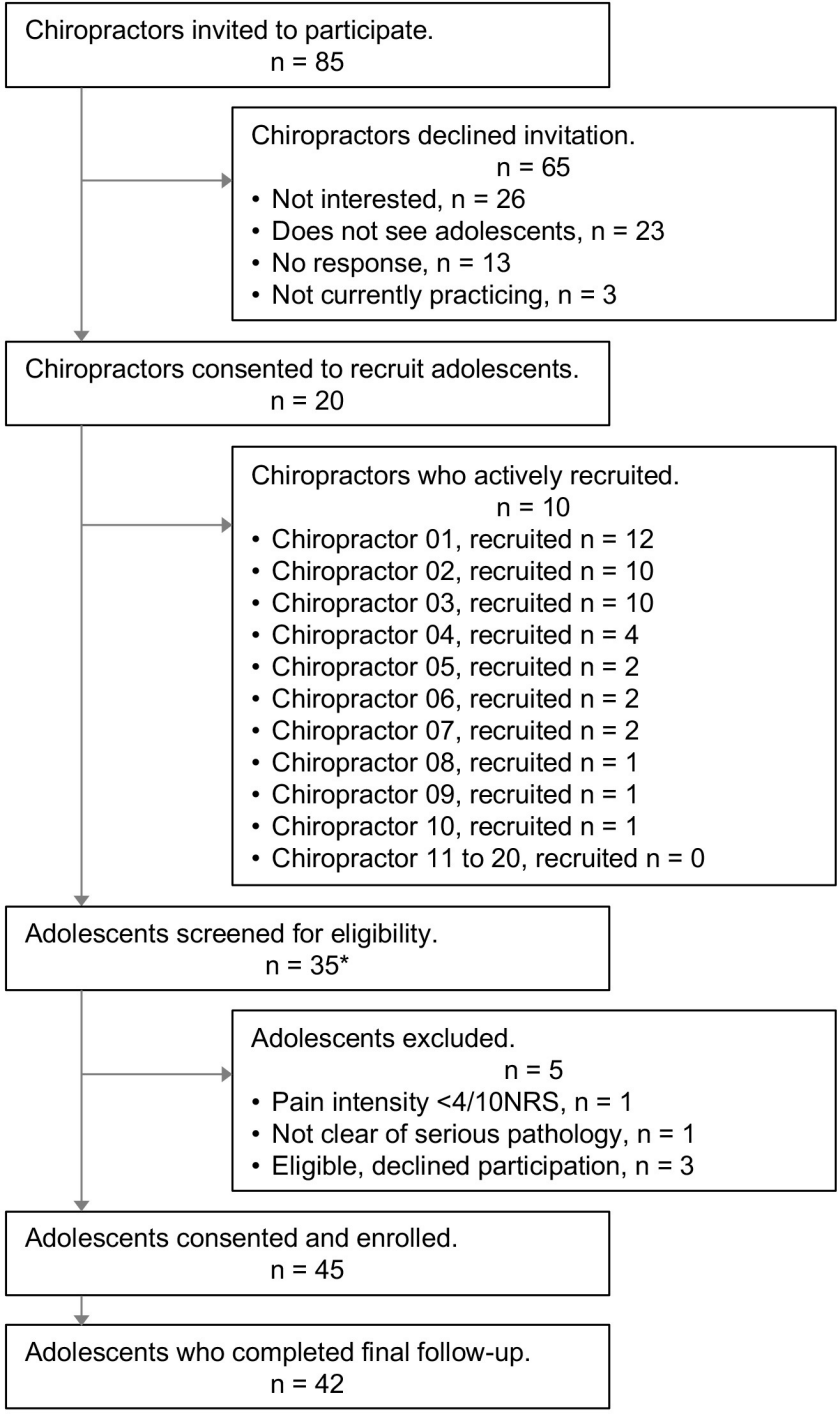
Chiropractor and adolescent flow through the study. *Successfully recorded number of adolescents screened.

We invited 85 chiropractors; 20 agreed to recruit, and 10 successfully recruited, four of whom recruited 80% of the 45 adolescents. Accurate records were *not* kept for the total number of consecutive adolescents who (1) presented for care, (2) were screened, (3) were deemed ineligible or (4) declined to participate with reasons. Only 35 screening forms were successfully recorded, 30 of which enrolled in the study. The flow of chiropractors and adolescents through the study is illustrated in figure 1.

Chiropractors began recruitment upon their consent; it took 11 months to recruit 20 chiropractors. Recruitment of 45 adolescents took 13.5 months, excluding an 8-month pause due to COVID-19 interruptions. On average, the overall *recruitment rate* of adolescents into the study was 3.3 per month. The average recruitment time for the 10 recruiting chiropractors was 7.2 months, with a recruitment rate of 0.6 adolescents per chiropractor per month.

The *response rates* and completion times for each data collection instrument are outlined in table 1. For Twilio compatibility, the mobile phone numbers for 33 participants (73%) had to be corrected to international format. The average response rate to all weekly SMSs was 94% (89%–98%). In 1% of all SMS data measures (n=10 of 990 total possible SMSs), adolescents actively returned empty data; these were not treated as non-responses. Phone reminders resulted in completing two full and two partial baseline questionnaires (n=4 of 45, 9%), 12 weekly SMSs (n=12 of 990, 1%) and no follow-ups. One-quarter (27%) of adolescents reported receiving assistance completing their baseline questionnaire, 17% with weekly SMSs and 14% with their final follow-up questionnaire.

**Table 1:** Response rate and time to complete for each data collection instrument

Data collection instrument	Response rate *(n/N)*		Time taken to complete median (range)ˆ	
Information and consent				
Adolescent	100% (45/45)		6 min 6 s (1 min 38 s to 3 hours 0 min 26 s)	
Chiropractor	100% (20/20)		2 min 16 s (53 s to 53 min 36 s)	
Baseline questionnaire				
Adolescent	97.8% (44/45)		9m36s (3m56s to 7h46m19s)	
Chiropractor	100% (45/45)		1m28s (33 s to 59m20s)	
Weekly SMS, adolescent	SMS (1) NRS	SMS (2) GBRS		
Week 1	97.8% (44/45)	93.3% (42/45)	–	–
Week 2	93.3% (42/45)	93.3% (42/45)	–	–
Week 3	95.6% (43/45)	93.3% (42/45)	–	–
Week 4	97.8% (44/45)	91.1% (41/45)	–	–
Week 5	93.3% (42/45)	93.3% (42/45)	–	–
Week 6	93.3% (42/45)	93.3% (42/45)	–	–
Week 7	95.6% (43/45)	95.6% (43/45)	–	–
Week 8	95.6% (43/45)	95.6% (43/45)	–	–
Week 9	93.3% (42/45)	93.3% (42/45)	–	–
Week 10	91.1% (41/45)	91.1% (41/45)	–	–
Week 11	88.9% (40/45)	88.9% (40/45)	–	–
Overall	93.5% (926/990)			
Follow-up questionnaire				
Adolescent	93.3% (42/45)		3 min 30 s (1 min 41 s to 19 min 41 s)	
Chiropractor	100% (45/45)		1 min 14 s (13 s to 22 hours 36 min 41 s)	

NRS; pain intensity via the , GBRS; pain recovery via the Global Back Rating Scale, ˆ Median (range) *=* complete median instrument completion time (shortest to longest completion time), h; hours, m; minutes, s; seconds.

* (n/N) = as a percentage of responses received (n) over total responses expected (N).

GBRSGlobal Back Rating ScaleNRSNumeric Rating Scale

The *retention rate* of adolescents at study completion was 93%. Three adolescents lost to follow-up had low response rates for all data collection instruments, ranging from 42% to 70%. One adolescent participant, not lost to follow-up, had very low responses to all data collection instruments (17%) yet still returned their final follow-up questionnaire. Due to the low number of participants lost to follow-up (n=3), we omitted a planned comparison with those who completed follow-up.

### Participants

The adolescent mean age was 15.4 years (±1.4, 12.6–17.9 years), and 43% were female. See online supplemental resource 2, table 2 for all adolescent and chiropractor baseline and follow-up measures and results.

At baseline, adolescents reported median pain intensity as 4.5/10 (IQR 4–6) in the past week and 4/10 (IQR 1–5) currently. Notably, 47% of adolescents at baseline reported a *current pain intensity* of <4/10, and 16% had an NRS score of <4 when reporting their *average pain intensity in the past week*; these adolescents may have been inappropriately enrolled. When asked how long they thought the resolution of their spinal pain would take, 14% of adolescents said 0–2 days, 32% 3–14 days, 23% 15–30 days, 21% 31–60 days and 11% >60 days. When asked how they expected their spinal pain to change in 3 months, 14% of adolescents expected no change, and 16% expected to be completely recovered. For 29%, there was a difference between the primary region of spinal pain reported by the adolescent and chiropractor. Chiropractors reported 38% of adolescents had experienced previous episodes of spinal pain, and 78% of cases had a non-traumatic mechanism of onset. Adolescents averaged over 60 min of exercise 4 days/week and engaged in 3 hours of screen time on school days, excluding schoolwork. Mean values and comparisons for PedsQL are available in online supplemental resource 2, table 3.

**Figure 2:**
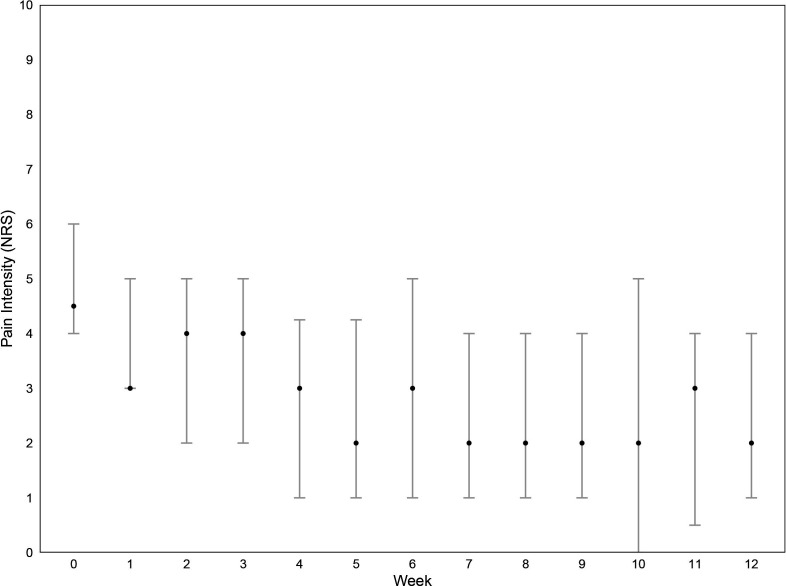
Spinal pain intensity over the past week. Median and IQR. Pain intensity (Numeric Rating Scale): 0=no pain, 10=worst possible pain. Missing: 7%, 34 missing responses from 585 possible responses (ie, 45 adolescents were to be assessed on 13 occasions).

Adolescents’ week-by-week pain intensity (NRS) and perceived pain recovery (GBRS) are shown in figures2  3. Mean pain intensity decreased in the first half of the study period before stabilising. There was no change in perceived pain recovery.

**Figure 3:**
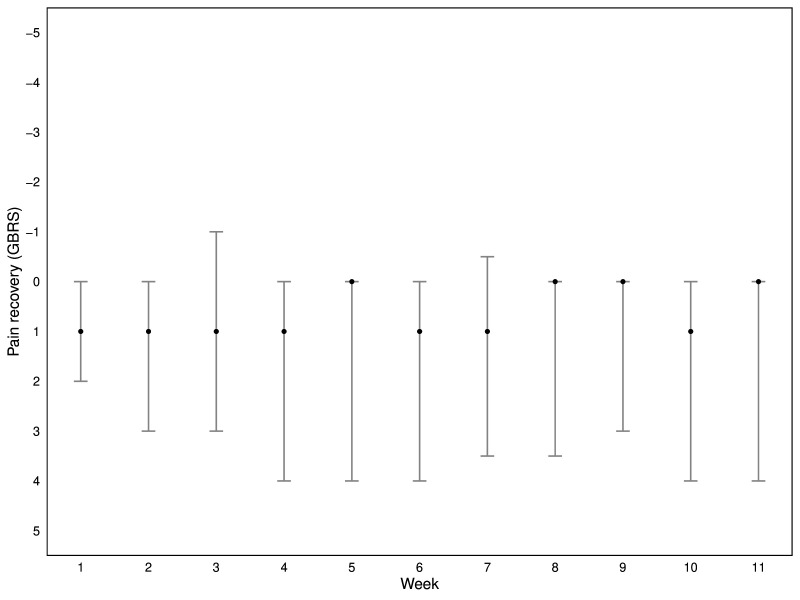
Percieved spinal pain recovery over the past week. Median and IQR. Pain recovery (Global Back Rating Scale); y-axis inverted, 5=completely recovered and −5=much worse. Missing: 8%, 11 missing responses from 495 possible responses (ie, 45 adolescents were to be assessed on 11 occasions).

At final follow-up, adolescents reported median pain intensity in the past week of 2/10 (IQR 1–4) and current as 2/10 (IQR 1–3), 19% reported no pain, and 45% reported mild pain intensity (<4/10 NRS). When asked to recall the past 3 months, 19% reported no change in pain, and 14% stated they had completely recovered. The median duration of care given to adolescents was two weeks, with a median of two appointments and a median cost of AU$190.00. Chiropractors referred 18% for imaging (X-ray: n=8, MRI: n=1). Five adolescents reported an adverse event from care, of which four reported the event lasted less than two days. The most common adverse event was fatigue/tiredness (n=4), followed by discomfort/pain (n=3), stiffness (n=2), weakness (n=2), difficulty sleeping (n=2), headache (n=1) and difficulty walking (n=1). No adolescents reported dizziness, numbness/tingling, nausea/vomiting or other adverse event.

### Recovery

**Figure 4:**
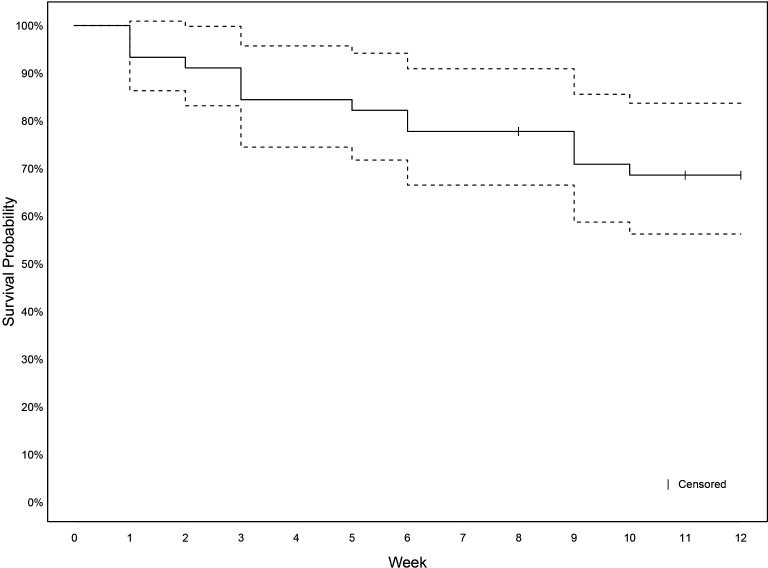
Kaplan-Meier survival plot for moderate-to-severe spinal pain episode recovery in adolescents.

Fourteen adolescents (31%) experienced recovery from their spinal pain episode during the 12-week study period (figure 4). Among these, nine (64%) reported a second episode of spinal pain after recovery from their initial episode before the study’s end.

## Discussion

### Feasibility

Our study demonstrated high *retention* and *response rates* among adolescents over the 12-week study period, supporting the feasibility of a large-scale cohort study. While SMS data collection was largely effective, the most significant challenge was ensuring mobile phone numbers were entered in the correct format. Future studies could address this issue by enforcing a restricted entry format rather than relying on written instruction alone. Minimal other issues arose; for example, one adolescent did not have international roaming enabled while overseas, and parental phone confiscation occurred for another adolescent. Our reminder protocols effectively mitigated these challenges.

Our *recruitment rate* highlights limitations in our approach. Projecting to recruit 1000 adolescents in a large-scale cohort could take 17 months with our existing approach. This would require inviting 850 chiropractors, 200 agreeing to participate and 100 ultimately recruiting adolescents. Though the challenge of recruitment from primary care settings is commonly encountered, we would need substantial refinements to mitigate the need for such large numbers of practitioners and to expedite recruitment.

### Limitations

Relying solely on chiropractors may not be feasible in a large-scale cohort. Only half the chiropractors who consented to recruit adolescents successfully recruited. Four chiropractors completed 80% of adolescent participant recruitment. Clinician agreement and willingness do not reliably predict actual participation.^24^ This may stem from difficulty identifying and enrolling eligible participants without disrupting clinical consultations. Anecdotal feedback indicated the screening and enrolment process were cumbersome, a sentiment echoed in the data. Consecutive adolescent screening data were not recorded successfully, so it is unknown if consecutive screening occurred, how many adolescents were screened, their eligibility status and reasons for non-participation. Also, nearly half the participants (47%) reported *current pain intensity* below the moderate-to-severe inclusion criterion at baseline. Baseline questionnaires were planned to be completed almost immediately after screening, though, in some cases, the adolescent completed their questionnaire after consultation. Thus, the care provided by their chiropractor may have decreased their pain intensity.

### Solutions

To address the problem of low recruitment, we propose expanding clinician involvement beyond chiropractors to include physiotherapists and general practitioners, as well as specialised clinics such as spinal pain or chronic pain centres. By identifying high care-seeking settings before commencement, we could strategically allocate resources to support higher-yield recruitment, that is, embed research staff in clinics to ensure consecutive and accurate screening, enrolment and timely collection of baseline data. Also, if we engage with practitioners earlier in planning study procedures, we can better understand and respond to the factors influencing active participation. To improve the accuracy of consecutive screening and data recording for ineligible patients, compensating clinicians for their time spent on screening and data entry should be strongly considered.^25^

### Spinal pain clinical course

One-third of participants recovered from their enrolment episode of spinal pain, of whom 64% experienced another episode before the study concluded. This aligns with Dissing *et al*’s findings, where one-quarter of 1,465 Danish children aged 8–14 years experienced three or more episodes of spinal pain annually over 3 years.^26^ Aartun *et al*’s 2-year longitudinal study of 1,042 Danish adolescents aged 12–15 similarly found that adolescents with higher intensity pain (>4/10 NRS, as in our cohort) experienced more frequent episodes.^27^ These findings emphasise the limitation of our 12-week study period in capturing the entire clinical course of moderate-to-severe spinal pain in adolescents and for identifying individuals at higher risk of chronic or recurrent spinal pain.

### Generalisability

The preliminary nature of this feasibility study warrants caution regarding generalisability. Several factors contributed to the non-representativeness of our sample, including recruitment methods (participants recruited from 10 chiropractors in Sydney, Australia), the study population (adolescents with moderate-to-severe spinal pain seeking chiropractic care) and study-specific challenges (limited data on potentially eligible participants). For instance, our sample had 43% females, which differs from previous spinal pain research, where females typically predominate.^28^ In a larger cohort, recruiting an Australian-wide sample from various healthcare settings would likely produce a more representative sample from diverse backgrounds, both rural and metropolitan areas and high- and low-socioeconomic backgrounds, supporting better generalisability.

### Interpretation

This study is the first prospective cohort of adolescent spinal pain conducted in Australian chiropractic care, making direct comparisons challenging. However, Swain *et al* conducted a clinical feasibility study on adolescents with knee pain, achieving a 71% SMS response and 80% retention rate at 6-month follow-up.^20^ Our study adopted proposed changes from Swain *et al*, such as automating follow-up measures, resulting in increased response rates and reduced costs. Additionally, we suggest combining the 2 weekly SMSs in a future cohort to improve response rates, minimise points of contact and half SMS-related expenses. Michaleff *et al*’s feasibility study in England used electronic health records to target adolescents with musculoskeletal conditions.^29^ While they achieved a 100% retention rate at a 6-week follow-up, their consent rate was below 20% of those invited and below 10% of those eligible, leading them to deem their approach unfeasible. They hypothesised that insufficient engagement with parents and guardians, who ultimately act as the decision-makers for young people’s health and participation in research, contributed to these findings. These varying feasibility outcomes emphasise the importance of understanding specific contexts and target populations when transitioning from feasibility to cohort studies.

### Conclusions

Our feasibility study’s high response and retention rates indicate the viability of our methods for a large-scale prospective cohort study exploring the clinical course of moderate-to-severe spinal pain in adolescents seeking chiropractic care. Refinements to address low recruitment and sample generalisability will be essential for the success of a larger study.

## supplementary material

10.1136/bmjopen-2024-088834 online supplemental file 1

10.1136/bmjopen-2024-088834 online supplemental file 2

## Data Availability

Data are available upon reasonable request.
