# Supplementary material for: Clinical course of spinal pain in adolescents: a feasibility study in a chiropractic setting
Source: BMJ Open. 2025 Jan 30;15(1):e088834. doi: 10.1136/bmjopen-2024-088834 (PMC11795366; doi:10.1136/bmjopen-2024-088834)
Supplement: online supplemental file 1 [file bmjopen-15-1-s001.docx]

**Online Resource One (of Two)**

**The clinical course of spinal pain in adolescents: a feasibility study in a chiropractic setting**

Laura RC Montgomery, Steven J Kamper, Anika Young, Amber Beynon, Katherine A Pohlman, Lise Hestbæk, Mark J Hancock, Simon D French, Christopher G Maher, Michael S Swain.

*BMJ Open*

**Data collection instrument 1. Adolescent Screening**

Chiropractor's full name:

Date:

Patient initials:

1. Is the patient presenting for care aged between 12 -17 years? Yes/No

ILLUSTRATION OF NECK PAIN, MID BACK PAIN AND LOW BACK PAIN HERE.

1. Is the patient presenting with a new episode of spinal pain - either neck, mid-back or low-back pain? Yes/No
2. Is the neck pain located in the region on the illustration above? (The pain may or may not radiate/refer to the extremities or head.) Yes/No
   1. Did the onset of this episode start within the past 6-weeks? Yes/No
   2. Is the neck pain of moderate-to-severe intensity right now, defined as 4/10 numerical rating scale? Yes/No
   3. Was the current episode of neck pain preceded by 1-month with no spinal pain (defined as 0/10 NRS)? Yes/No

OR

Is the mid-back pain located in the region illustrated in the diagram above? (The pain may or may not radiate/refer to the extremities or head.) Yes/No

1. Did the onset of this episode start within the past 6-weeks? Yes/No
2. Is the mid-back pain of moderate-to-severe intensity right now, defined as 4/10 numerical rating scale? Yes/No
3. Was the current episode of mid-back pain preceded by 1-month with no spinal pain (defined as 0/10 NRS)? Yes/No

OR

Is the low-back pain located in the region illustrated in the diagram above? (The pain may or may not radiate/refer to the extremities.) Yes/No

- 1. Did the onset of this episode start within the past 6-weeks? Yes/No
  2. Is the low-back pain of moderate-to-severe intensity right now, defined as 4/10 numerical rating scale? Yes/No
  3. Was the current episode of low-back pain preceded by 1-month with no spinal pain (defined as 0/10 NRS)? Yes/No

1. Is the patient able to speak and read English? Yes/No
2. Does the patient own a smart phone with internet and SMS functionality? Yes/No
3. Can the patient respond to text messages and email? Yes/No
4. Is the patient clear of whiplash associated disorder and serious spinal pathology (e.g. fracture, infection)? Yes/No
5. Has the patient and their parent (or guardian) read and understood the participant information, and are willing to consent to participate in the study? Yes/No

Unfortunately the patient is not eligible to be included in The COURSE Study.

OR

The patient is eligible to participate in The COURSE Study! Please ask the patient to scan the following QR code to be directed to the patient information and consent form. They will need to fill this out with their parent/guardian.

**Data collection instrument 2. Adolescent Baseline**

Please complete the following baseline survey. This survey should take less than 30 minutes to complete.

You can use the plus and minus icon in the top right-hand corner of your display to adjust the size of the text.

If you need to return to the previous page, please use the 'Back' button at the bottom of the screen. Do not use the back arrow in the browser.

If you cannot complete the survey at this time, please click on the 'Save & return later' button below.

Thank you for your time.

**Demographics**

1. Postal address (Please include full street address and postcode)
2. Date of birth
3. Sex: Male/Female
4. Age
5. Year in school?

**Spinal Pain Characteristics**

- - 1. This questionnaire is related to your neck, mid-back or low back pain, which we refer to as "spinal pain"

ILLUSTRATION OF NECK PAIN, MID BACK PAIN AND LOW BACK PAIN HERE.

Right now, what is the primary location of your spinal pain, that is, what is the main location of your pain? (Use the diagram above) neck pain/mid-back pain/low back pain

1. What is your level of neck pain right now? 0 no pain 1 2 3 4 5 6 7 8 9 10 worst pain imaginable
2. What was your typical level of neck pain intensity over the past week? 0 no pain 1 2 3 4 5 6 7 8 9 10 worst pain imaginable

OR

1. What is your level of mid-back pain right now? 0 no pain 1 2 3 4 5 6 7 8 9 10 worst pain imaginable
2. What was your typical level of mid-back pain intensity over the past week? 0 no pain 1 2 3 4 5 6 7 8 9 10 worst pain imaginable

OR

1. What is your level of low back pain right now? 0 no pain 1 2 3 4 5 6 7 8 9 10 worst pain imaginable
2. What was your typical level of low back pain intensity over the past week? 0 no pain 1 2 3 4 5 6 7 8 9 10 worst pain imaginable
   - 1. How do you expect your >BACK PAIN< will change in 3-months?-5 very much worse -4 -3 -2 -1 0 unchanged 1 2 3 4 5 completely recovered
     2. What length of time do you expected for resolution of your spinal pain: 0 to 2 days 3 to 7 days 8 to 14 days 15 to 30 days 31 to 60 days more than 60 days
     3. Over the past week, on how many days did you take medication for your spinal pain? 0 days 1 2 3 4 5 6 7 days

**Pain Coping**

Here is a list of things that teenagers sometimes do when they are in pain. For each of the choices, please indicate if you do it never, sometimes, or often by selecting the choice in the column on the right. Please be sure to select a response for each item.

When I feel pain I: Never/Sometimes/Often

1. Go to bed
2. Ask for medicine
3. Ask for a hug or kiss
4. Ask for someone to understand my pain
5. Cry or yell
6. Think about going away on vacation or a trip
7. Visit with my friends
8. Watch TV
9. Play a game
10. Eat or drink something
11. Rub the sore spot
12. Tell myself I can handle it
13. Have a parent or friend sit with me
14. Try not to think about the pain or ignore the pain
15. Breathe deeply
16. Think about happy things
17. Play with my pet
18. Read a book
19. Talk about what I did today
20. Think it will just get worse
21. Wish for it to go away
22. Imagine I can make the pain disappear by myself
23. Pretend I don't have any pain

**Quality of Life**

Below is a list of things that might be a problem for you. Please tell us how much of a problem each one has been for you during the past 7 days.

About my health and activities (problems with spinal pain) Never/Almost never/Sometimes/Often/Almost always

1. It is hard for me to walk more than one block
2. It is hard for me to run
3. It is hard for me to do sports activities or exercise
4. It is hard for me to lift something heavy
5. It is hard for me to take a bath or shower by myself
6. It is hard for me to do chores around the house
7. I hurt or ache
8. I have low energy

About my feelings (problems with spinal pain) Never/Almost never/Sometimes/Often/Almost always

1. I feel afraid or scared
2. I feel sad or blue
3. I feel angry
4. I have trouble sleeping
5. I worry about what will happen to me

How I get along with others (problems with spinal pain) Never/Almost never/Sometimes/Often/Almost always

1. I have trouble getting along with other teens
2. Other teens do not want to be my friend
3. Other teens tease me
4. I cannot do things that other teens my age can do
5. It is hard to keep up with my peers

About school (problems with spinal pain) Never/Almost never/Sometimes/Often/Almost always

1. It is hard to pay attention in
2. I forget things
3. I have trouble keeping up with my schoolwork
4. I miss school because of not feeling well
5. I miss school to go to the doctor or hospital

**Fatigue**

Below is a list of things that might be a problem for you. Please tell us how much of a problem each one has been for you during the past 7 days.

General fatigue (problems with spinal pain) Never/Almost never/Sometimes/Often/Almost always

1. I feel tired
2. I feel physically weak
3. I feel too tired to do things that I like to do
4. I feel too tired to spend time with my friends
5. I have trouble finishing things
6. I have trouble starting things

Sleep/Rest Fatigue (problems with spinal pain) Never/Almost never/Sometimes/Often/Almost always

1. I sleep a lot
2. It is hard for me to sleep through the night
3. I feel tired when I wake up in the morning
4. I rest a lot
5. I take a lot of naps
6. I spend a lot of time in bed

Cognitive fatigue (problems with spinal pain) Never/Almost never/Sometimes/Often/Almost always

1. It is hard for me to keep my attention on things
2. It is hard for me to remember what people tell me
3. It is hard for me to remember what I just heard
4. It is hard for me to think quickly
5. I have trouble remembering what I was just thinking
6. I have trouble remembering more than one thing at a time

During the past 7 days, on how many days were you physically active for a total of at least 60 minutes per day? (Add

up all the time you spent in any kind of physical activity that increased your heart rate and made you breathe hard

some of the time.) 0 days/1 days/2 days/3 days/4 days/5 days/6 days/7 days

On an average school day, how many hours do you spend in front of a TV, computer, smart phone, or other electronic

device watching shows or videos, playing games, accessing the internet, or using social media (also called "screen

time")? (Do not count time spent doing schoolwork.) Less than 1 hour per day/1 hour per day/2 hours per day/3 hours per day/4 hours per day/5 or more hours per day

In an average week when you are in school, on how many days do you go to physical education (PE) classes? 0 days/1 2/3/4/5 days

During the past 12 months, in how many sports teams did you play? (Count any teams run by your school or

community groups.) 0 teams/1 team/2 teams/3 or more teams

Did you receive assistance in completing this form? Yes/No

If completing this survey has raised any negative emotions for you please contact:

Kids help line 1800 55 1800 | website https://kidshelpline.com.au/

OR

Lifeline 13 11 14 | website https://www.lifeline.org.au/

OR

Your doctor

**Data collection instrument 3. Chiropractor Baseline**

Please complete the form below for >FIRST NAME< >LAST NAME<

The date of the initial visit was > DATE<

ILLUSTRATION OF NECK PAIN, MID BACK PAIN AND LOW BACK PAIN HERE.

1. What is the primary region of the patient's spinal pain right now? Neck pain/Mid-back pain/Low back pain
2. Does the spinal pain radiate/refer to the extremities and/or head? Yes/No
3. Does the primary spinal pain coexist with other pain conditions? (Select all regions that coexist) Neck Mid-back Low-back Head/Face Upper extremity Lower extremity Chest Abdomen Other None
4. Please specify other:
5. How many weeks ago did the current episode of spinal pain begin? <1 week/1/2/3/4/5/>5 weeks
6. What was the mechanism of injury that incited the current episode of spinal pain? Trauma/Non-trauma/Unclear
7. Has the patient experienced this type of spinal pain in the past? Yes/No
8. Please describe how many months/years ago did the first episode of spinal pain begin? (In whole months/years)
9. How many occasions has this type of spinal pain reoccurred?(in whole numbers)
10. Has the patient received chiropractic care for this type of spinal pain in the past? Yes/No
11. Please report on the presence of hypermobility, using Beighton's scoring method below.
    1. Passive hyperextension of the elbow beyond 10° Left Right Neither
    2. Passive dorsiflexion and hyperextension of the fifth MCP joint beyond 90° Left Right Neither
    3. Passive apposition of the thumb to the flexor aspect of the forearm Left Right Neither
    4. Passive hyperextension of the knee beyond 10° Left Right Neither
    5. Active forward flexion of the trunk with the knees fully extended so that the palms of the hands rest flat on the floor? Yes
12. Please report on the presence of hypomobility, using the Fingertips to Floor Distance Test. The patient is asked to bend forward and attempt to reach for the floor with their fingertips.
    1. What is the distance between the patient's middle finger and the floor using a standard measuring tape? (please report in cm)
    2. Did pain, stiffness or both limited the Fingertips to floor (FTF) test? Pain/stiffness/test not limited

**Data collection instrument 4. Adolescent Weekly SMS**

**SMS 1**

On a number scale of 0 to 10 (0=no pain, 10=worst possible pain). Over the PAST WEEK, what was your TYPICAL level of spinal pain? 0 no pain 1 2 3 4 5 6 7 8 9 10 worst pain imaginable

**SMS 2**

On a number scale of -5 to 5 (-5=Very much worse, 0=No change, 5=Completely recovered). Please rate the extent of your RECOVERY from spinal pain, over the PAST WEEK. -5 very much worse -4 -3 -2 -1 0 unchanged 1 2 3 4 5 completely recovered

**Data collection instrument 5. Adolescent Follow-up**

Please complete the following survey. This survey should take less than 15 minutes to complete. You can use the

plus and minus icon in the top right-hand corner of your display to adjust the size of the text.

If you need to return to the previous page, please use the 'Back' button at the bottom of the screen. Do not use the

back arrow in the browser.

If you cannot complete the survey at this time, please click on the 'Save & return later' button below.

Thank you for your time.

**Spinal Pain Characteristics**

1. This questionnaire is related to your neck, mid-back or low back pain, which we refer to as "spinal pain"
2. What is your level of >BACK PAIN< right now? 0 no pain 1 2 3 4 5 6 7 8 9 10 worst pain imaginable
3. What was your typical level of >BACK PAIN< intensity over the past week? 0 no pain 1 2 3 4 5 6 7 8 9 10 worst pain imaginable
4. With respect to your >BACK PAIN<, how are you today compared to when you first completed the >BACK PAIN< questionnaires 3-months ago? (please select your choice) -5 very much worse -4 -3 -2 -1 0 unchanged 1 2 3 4 5 completely recovered
5. Over the past week, on how many days did you take medication for your spinal pain? 0 days 1 2 3 4 5 6 7 days

**Pain Coping**

Here is a list of things that teenagers sometimes do when they are in pain. For each of the choices, please indicate if you do it never, sometimes, or often by selecting the choice in the column on the right. Please be sure to select a response for each item.

When I feel pain I: Never/Sometimes/Often

1. Go to bed
2. Ask for medicine
3. Ask for a hug or kiss
4. Ask for someone to understand my pain
5. Cry or yell
6. Think about going away on vacation or a trip
7. Visit with my friends
8. Watch TV
9. Play a game
10. Eat or drink something
11. Rub the sore spot
12. Tell myself I can handle it
13. Have a parent or friend sit with me
14. Try not to think about the pain or ignore the pain
15. Breathe deeply
16. Think about happy things
17. Play with my pet
18. Read a book
19. Talk about what I did today
20. Think it will just get worse
21. Wish for it to go away
22. Imagine I can make the pain disappear by myself
23. Pretend I don't have any pain

**Quality of Life**

Below is a list of things that might be a problem for you. Please tell us how much of a problem each one has been for you during the past 7 days.

About my health and activities (problems with spinal pain) Never/Almost never/Sometimes/Often/Almost always

1. It is hard for me to walk more than one block
2. It is hard for me to run
3. It is hard for me to do sports activities or exercise
4. It is hard for me to lift something heavy
5. It is hard for me to take a bath or shower by myself
6. It is hard for me to do chores around the house
7. I hurt or ache
8. I have low energy

About my feelings (problems with spinal pain) Never/Almost never/Sometimes/Often/Almost always

1. I feel afraid or scared
2. I feel sad or blue
3. I feel angry
4. I have trouble sleeping
5. I worry about what will happen to me

How I get along with others (problems with spinal pain) Never/Almost never/Sometimes/Often/Almost always

1. I have trouble getting along with other teens
2. Other teens do not want to be my friend
3. Other teens tease me
4. I cannot do things that other teens my age can do
5. It is hard to keep up with my peers

About school (problems with spinal pain) Never/Almost never/Sometimes/Often/Almost always

1. It is hard to pay attention in
2. I forget things
3. I have trouble keeping up with my schoolwork
4. I miss school because of not feeling well
5. I miss school to go to the doctor or hospital

**Chiropractic care**

1. Considering pain relief, side effects, physical recovery, emotional recovery, and economic considerations (if appropriate), how satisfied were you with the chiropractic care you received? 1 Completely satisfied, couldn't be better 2 3 4 5 6 7 Completely dissatisfied, couldn't be worse
2. An adverse event is any unfavourable sign, symptom, or disease temporarily associated with the chiropractic treatment, whether or not caused by the treatment, specifically any worsened or new symptom up to 1 week after care.
   1. In the past 3-months did you experience any adverse events following chiropractic care? Yes/No
   2. Please describe the adverse event(s): (select all that apply) Discomfort or pain/Stiffness/Weakness / Fatigue or tiredness/Headache/Dizziness/Numbness or tingling/Nausea or vomiting/Difficulty walking/Problems sleeping/Other
   3. Please specify other:
   4. How long did it take for resolution of adverse events? 0 to 2 days/3 to 7 days/8 to 14 days/15 to 30 days/31 to 60 days/more than 60 days
   5. As a result of adverse events from chiropractic care did you:
      1. Seek care from a medical doctor? Yes/No
      2. Hospital care? Yes/No

Did you receive assistance in completing this form? Yes/No

Over the past 11-weeks, did you receive assistance in completing any of the text message questions? Yes/No

If completing this survey has raised any negative emotions for you please contact:

Kids help line 1800 55 1800 | website https://kidshelpline.com.au/

OR

Lifeline 13 11 14 | website https://www.lifeline.org.au/

OR

Your doctor

**Data collection instrument 6. Chiropractor Follow-up**

This survey report seeks information about the clinical care of >FIRST NAME< >LAST NAME< over the past 3-months.

Please use your clinical notes to complete this form.

1. Regarding the duration of care, how many weeks was the period that this patient received chiropractic care?
2. What was the total number of patient visits for chiropractic care?
3. What was the total cost of chiropractic care in the 3-month period?
4. Was this patient referred for imaging? Yes/No
5. What type of imaging? X-ray/MRI/CT/Ultrasound/Other
6. Please specify other:
7. In the text box below, please name the types of interventions used to treat the patient's spinal pain. Please rank

order these interventions from most frequently used to least frequently used. (Please separate by comma, please don't use acronyms or shorthand.)
