# Supplementary material for: Clinical course of spinal pain in adolescents: a feasibility study in a chiropractic setting
Source: BMJ Open. 2025 Jan 30;15(1):e088834. doi: 10.1136/bmjopen-2024-088834 (PMC11795366; doi:10.1136/bmjopen-2024-088834)
Supplement: online supplemental file 2 [file bmjopen-15-1-s002.docx]

**Online Resource Two (of Two)**

**The clinical course of spinal pain in adolescents: a feasibility study in a chiropractic setting**

Laura RC Montgomery, Steven J Kamper, Anika Young, Amber Beynon, Katherine A Pohlman, Lise Hestbæk, Mark J Hancock, Simon D French, Christopher G Maher, Michael S Swain.

*BMJ Open*

**Table 2** Descriptive statistics

|  | **Baseline Questionnaire** | | **Follow-up Questionnaire** | |
| --- | --- | --- | --- | --- |
|  | **Adolescent** | **Chiropractor** | **Adolescent** | **Chiropractor** |
| **Spinal Pain Variables** |  |  |  |  |
| SP primary region, *n(%)* |  |  |  |  |
| Neck | 17 (38.6%) | 16 (35.6%) | - | - |
| Mid-back | 8 (18.2%) | 16 (35.6%) | - | - |
| Low back | 19 (43.2%) | 13 (28.9%) | - | - |
| SP NRS last week, *median(IQR)* | 4.5 (4 to 6) | - | 2 (1 to 4) | - |
| SP NRS now, *median(IQR)* | 4 (1 to 5) | - | 2 (1 to 3) | - |
| SP recovery, *n(%)* | *Expected change in next 3 months* |  | *Reflective change in last 3 months* |  |
| -5 Very much worse | 0 (0%) | - | 0 (0%) | - |
| -4 | 0 (0%) | - | 0 (0%) | - |
| -3 | 1 (2.3%) | - | 1 (2.4%) | - |
| -2 | 2 (4.7%) | - | 1 (2.4%) | - |
| -1 | 2 (4.7%) | - | 2 (4.8%) | - |
| 0 No change | 6 (14.0%) | - | 8 (19.1%) | - |
| 1 | 7 (16.3%) | - | 8 (19.1%) | - |
| 2 | 8 (18.6%) | - | 6 (14.3%) | - |
| 3 | 7 (16.3%) | - | 3 (7.1%) | - |
| 4 | 3 (7.0%) | - | 7 (16.7%) | - |
| 5 Completely recovered | 7 (16.3%) | - | 6 (14.3%) | - |
| SP episode expected duration, *n(%)* |  |  |  |  |
| 0-2 days | 6 (13.6%) | - | - | - |
| 3-7 days | 8 (18.2%) | - | - | - |
| 8-14 days | 6 (13.6%) | - | - | - |
| 15-30 days | 10 (22.7%) | - | - | - |
| 31-60 days | 9 (20.5%) | - | - | - |
| >60 days | 5 (11.4%) | - | - | - |
| SP refers to limbs/head, yes: *n(%)* | - | 8 (17.8%) | - | - |
| Coexisting pain, yes: *n* |  |  |  |  |
| Neck | - | 14 | - | - |
| Mid-back | - | 10 | - | - |
| Low back | - | 5 | - | - |
| Lower limb | - | 8 | - | - |
| Head | - | 3 | - | - |
| Upper limb | - | 0 | - | - |
| Chest | - | 0 | - | - |
| Abdomen | - | 0 | - | - |
| None | - | 15 | - | - |
| SP episode onset, *n(%)* |  |  |  |  |
| < 1week | - | 4 (8.9%) | - | - |
| 1 week | - | 5 (11.1%) | - | - |
| 2 weeks | - | 11 (24.4%) | - | - |
| 3 weeks | - | 5 (11.1%) | - | - |
| 4 weeks | - | 11 (24.4%) | - | - |
| 5 weeks | - | 1 (2.2%) | - | - |
| > 5 weeks | - | 8 (17.8%) | - | - |
| SP mechanism of onset, *n(%)* |  |  |  |  |
| Trauma | - | 4 (8.9%) | - | - |
| Non-trauma | - | 35 (77.8%) | - | - |
| Unclear | - | 6 (13.3%) | - | - |
| SP previous episodes, yes: *n(%)* | - | 17 (37.8%) | - | - |
| If yes; number of episodes, *median(range)* | - | 1 (0 to 12) | - | - |
| If yes, time since first episode | - | **Data unusable* | - | - |
| **PedsQL Pain Coping Inventory,** mean±SD(range) | 0.84±0.28 (0.22to1.39) |  | 0.71±0.27 (0.17to1.43) |  |
| **PedsQL Generic Core,** *mean±SD(range)* | 76±14(50to100) |  | 81±15(48to100) |  |
| Physical Functioning Domain | 70±15(31to100) | - | 76±19(28to100) | - |
| Emotional Functioning Domain | 70±20(35to100) | - | 80±19(35to100) | - |
| Social Functioning Domain | 88±15(50to100) | - | 89±14(50to100) | - |
| School Functioning Domain | 74±19(30to100) | - | 79±19(30to100) | - |
| **PedsQL Multidimensional Fatigue,** *mean±SD(range)* | 65±16(24to96) |  |  |  |
| General Fatigue Domain | 63±20(4to100) | - | - | - |
| Sleep/Rest Fatigue Domain | 60±16(21to96) | - | - | - |
| Cognitive Fatigue Domain | 71±20(25to100) | - | - | - |
| **Physical activity and sedentary behaviour variables** | |  |  |  |
| Days achieved 60min physical activity in last week, *mean±SD(range)* | 4.2±2.4 (0 to 7) | - | - | - |
| Days Physical Education classes in school week, *median(range)* | 2 (0 to 5) | - | - | - |
| Number of sports teams in last year, *median(range)* | 2 (0 to 3) | - | - | - |
| Screentime hours, not schoolwork, on average school day, *mean±SD(range)* | 3.3±1.3(0 to 5) | - | - | - |
| **Mobility Variables** |  |  |  |  |
| Beighton's Hypermobility, *median(range)* | - | 1 (0 to 9) | - | - |
| Normal: 0-3/9, *n(%)* |  | 32 (71.1%) |  |  |
| GJH: ≥4/9, *n(%)* [1] |  | 13 (28.9%) |  |  |
| Fingertip-to-floor distance, cm, *median(range)* | - | 2 (0 to 31) | - | - |
| Limited by pain | - | 3 | - | - |
| Limited by stiffness | - | 18 | - | - |
| **Healthcare Variables** |  |  |  |  |
| Number of days pain medication needed in last week, *median(range)* | 0 (0 to 4) | - | 0 (0 to 2) | - |
| Previous chiropractor care, yes: *n(%)* | - | 23 (51.1%) | - | - |
| Level of satisfaction with care |  |  |  |  |
| 1 Completely satisfied, couldn't be better | - | - | 21 (50%) | - |
| 2 | - | - | 13 (31.0%) | - |
| 3 | - | - | 4 (9.5%) | - |
| 4 | - | - | 1 (2.4%) | - |
| 5 | - | - | 1 (2.4%) | - |
| 6 | - | - | 2 (4.8%) | - |
| 7 Completely dissatisfied, couldn't be worse | - | - | 0 (0.0%) | - |
| Adverse event in last 3 months due to care, yes: *n(%)* | - | - | 5 (11.9%) | - |
| If yes, adverse event type, *n* |  |  |  |  |
| Discomfort/pain | - | - | 3 | - |
| Stiffness | - | - | 2 | - |
| Weakness | - | - | 2 | - |
| Fatigue/tiredness | - | - | 4 | - |
| Headache | - | - | 1 | - |
| Dizziness | - | - | 0 | - |
| Numbness/tingling | - | - | 0 | - |
| Nausea/vomiting | - | - | 0 | - |
| Difficulty walking | - | - | 1 | - |
| Problem sleeping | - | - | 2 | - |
| Other: | - | - | 0 | - |
| If yes, adverse event duration, *n* |  |  |  |  |
| 0-2 days | - | - | 4 | - |
| 3-7 days | - | - | 0 | - |
| 8-14 days | - | - | 0 | - |
| 15-30 days | - | - | 0 | - |
| 31-60 days | - | - | 0 | - |
| >60 days | - | - | 1 | - |
| If yes, adverse event care sought, *n* |  |  |  |  |
| Hospital | - | - | 0 | - |
| Medical doctor | - | - | 2 | - |
| Care duration, weeks, *median(range)* | - | - | - | 2 (1 to 20) |
| Number of visits, *median(range)* | - | - | - | 2 (1 to 12) |
| Care total cost, AU$, *median(range)* | - | - | - | 190 (70 to 1,000) |
| Imaging; yes, *n(%)* | - | - | - | 8 (17.8%) |
| X-ray, n | - | - | - | 5 |
| EOS, n | - | - | - | 3 |
| MRI, n | - | - | - | 1 |
| CT, n | - | - | - | 0 |
| US, n | - | - | - | 0 |
| Other, n | - | - | - | 0 |
| Chiropractic treatment modalities used | - | - | - | Activator, active release technique, advice (exercise, posture, rest), blocking (orthopaedic), cold laser, craniosacral therapy, diversified chiropractic adjustments, extremity manipulation/ mobilisation, high-velocity low-amplitude adjustment, massage machine, mobilisations (rotation, traction), post-isometric relaxation, rehabilitation exercises, remedial massage, sacro-occipital technique, soft tissue techniques, spinal manipulative therapy, stretching, stretches. |
| Legend: AU$; Australian dollar, cm; centimeters, CT; computed tomography, EOS; low-dose, weight-bearing X-ray technology, GJH; generalized joint hypermobility, mean±SD(range); mean, standard deviation and range for normally distributed data, median(range); median and full range for skewed data, median(IQR); median and interquartile range for skewed data, MRI; magnetic resonance imaging, n(%); number of participants and percentage of sample, NRS; numeric rating scale, PedsQL; Pediatric Quality of Life, SP; spinal pain, US; ultrasound, X-ray; radiograph.  Missing data, *n(%)*: Adolescent baseline; 1(2.2%), Chiropractor baseline; 0(0%), Adolescent follow-up; 3(6.7%), Chiropractor follow-up; 0(0%). | | | | |

**Table 3** PedsQL comparative data

|  | **COURSE**  **Baseline** | **COURSE**  **Follow-up** | **Nicola et al 2018 [2]** | **Gopinath et al 2013 [3]** | **Williams et al. 2005 [4]** | **Varni et al. 2001 [5]** | **Limpberg et al 2014 [6]** |
| --- | --- | --- | --- | --- | --- | --- | --- |
| ***Cohort*** | *Australian clinical cohort,*  *average age 15.4±1.4yr, acute pain group* | | *Australian control group, average age 8.8±2.9yrs* | *Australian students,*  *average age 17.3±0.5yrs, normal weight group* | *Australian students, average age 10.4±1.1yrs, standard weight group* | *American normative data,*  *average age 10.8±3.6yrs* | *Danish normative data,*  *average age 24.8±3.8yrs* |
| **PedsQL Generic Core,** mean±SD | 76±14 | 81±15 | 85.1±8.3 | M 78.6±0.7  F 82.5±2.1 | 80.5±12.2 | C 77.2±15.6  A 78.7±14.0  H 83.0±14.8 | C 76.7±15.9  H 85.9±11.5 |
| Physical Functioning | 70±15 | 76±19 | 82.4±9.9 | M 87.3±0.9  F 91.2±2.1 | 85.7±12.4 | C 77.4±20.4  A 78.9±19.1  H 84.4±17.3 | C 78.0±21.9  H 89.6±13.0 |
| Emotional Functioning | 70±20 | 80±19 | 77.2±14.3 | M 69.8±1.1  F 79.8±3.3 | 73.2±17.5 | C 76.4±21.5  A 77.3±20.0  H 80.9±19.6 | C 72.3±18.9  H 78.5±17.6 |
| Social Functioning | 88±15 | 89±14 | 87.0±13.2 | M 91.5±0.8  F 89.3±2.7 | 82.7±16.7 | C 81.6±20.2  A 82.8±16.7  H 87.4±17.2 | C 81.2±17.1  H 88.8±13.3 |
| School Functioning | 74±19 | 79±19 | 82.9±12.4 | M 63.7±1.2  F 66.7±4.1 | 77.1±15.4 | C 73.4±19.6  A 75.7±18.0  H 78.6±20.5 | C 74.5±18.0  H 84.4±14.4 |
| Legend: M, male; F, female; C, chronic health condition; A, acute health condition; H, healthy. | | | | | | | |

**References**

1. Malek S, Reinhold EJ, Pearce GS [2021] The Beighton Score as a measure of generalised joint hypermobility. Rheumatol Int 41:1707-1716. doi: 10.1007/s00296-021-04832-4

2. Nicola K, Watter P [2018] The comparison of perceived health-related quality of life between Australian children with severe specific language impairment to age and gender-matched peers. BMC Pediatr 18:62. doi: 10.1186/s12887-018-1058-2

3. Gopinath B, Baur LA, Burlutsky G, Mitchell P [2013] Adiposity adversely influences quality of life among adolescents. The Journal of adolescent health : official publication of the Society for Adolescent Medicine 52:649-653. doi: 10.1016/j.jadohealth.2012.11.010

4. Williams J, Wake M, Hesketh K, Maher E, Waters E [2005] Health-related quality of life of overweight and obese children. JAMA 293:70-76. doi: 10.1001/jama.293.1.70

5. Varni JW, Seid M, Kurtin PS [2001] PedsQL 4.0: reliability and validity of the Pediatric Quality of Life Inventory version 4.0 generic core scales in healthy and patient populations. Med Care 39:800-812. doi: 10.1097/00005650-200108000-00006

6. Limperg PF, Haverman L, van Oers HA, van Rossum MA, Maurice-Stam H, Grootenhuis MA [2014] Health related quality of life in Dutch young adults: psychometric properties of the PedsQL generic core scales young adult version. Health Qual Life Outcomes 12:9. doi: 10.1186/1477-7525-12-9
