# Supplementary material for: Clinical course of spinal pain in adolescents: a feasibility study in a chiropractic setting
Source: BMJ Open. 2025 Jan 30;15(1):e088834. doi: 10.1136/bmjopen-2024-088834 (PMC11795366; doi:10.1136/bmjopen-2024-088834)
Supplement: Supplementary file 3 [file bmjopen-2024-088834.reviewer_comments.pdf]

PEER REVIEW HISTORY

BMJ Open publishes all reviews undertaken for accepted manuscripts. Reviewers are asked to complete a checklist review form and are provided with free text boxes to elaborate on their assessment. These free text comments are reproduced below.

ARTICLE DETAILS

Title (Provisional)

The clinical course of spinal pain in adolescents: a feasibility study in a chiropractic setting

Authors

Montgomery, Laura; Kamper, Steven J; Young, Anika; Beynon, Amber; Pohlman, Katherine A.; Hestbæk, Lise; Hancock, Mark; French, Simon; Maher, Chris; Swain, Michael

---

VERSION 1 - REVIEW

**Reviewer**	**1**
**Name**	**Ben-Ami, Noa**
**Affiliation**	**Ariel University, Faculty of Health Sciences**
**Date**	**16-Nov-2024**
**COI**	**None**

This paper presents an important feasibility study addressing a critical issue, as chronic or recurrent back pain affects approximately 25% of adolescents and has a significant impact on their quality of life.

Background: The background is concise and well-articulated. However, it would benefit from a brief mention of how adolescent back pain may differ in nature, progression, or response to treatment compared to adults.

Methods:

Line 76-77: The sentence, “were invited to participate until 20 had consented to recruit adolescents,” is unclear. Please consider rephrasing for clarity.

Line 80: 45 adolescents overall?

Line 94: The adolescents got chiropractors' usual clinical care. So, it will be the clinical course of spinal pain in adolescents with chiropractors' usual clinical care. I think it will be good if you have a control group with no treatment to see the usual clinical course of adolescent back pain. for your consideration.

Line 174: It is surprising that the sample did not follow the typical gender distribution observed in similar studies, where females often outnumber males. This anomaly warrants further discussion.

Line 212 – Recovery rates appear notably low, with only 31% of adolescents recovering after six weeks. In contrast, the recovery rate in adults is typically around 70%. It will be good for discussion.

The discussion is thorough and provides valuable insights. The limitations are well-recognized, adding transparency and credibility to the study.

**Reviewer**	**2**
**Name**	**Hahne, Andrew**
**Affiliation**	**La Trobe University, Physiotherapy**
**Date**	**01-Dec-2024**
**COI**	**None**

This feasibility study is of interest as the population (adolescents with spinal pain) is not widely studied and in fact most clinical trials exclude this population. With some minor revisions the paper could better achieve its purpose of informing the feasibility of future research in this area.

Overall, the high rate of follow-up and retention was promising. Concerns with recruitment rate and sample representativeness are declared in the paper, although strategies to avoid them in a larger study could be more concrete.

Specific suggestions for improving the reporting of this study:

1. The setting of the study (chiropractic care) should be mentioned in the title, objectives and conclusions. This is often labelled in the article too generally as “Australian primary care”, of which the chiropractic setting represents only a small proportion. Adolescents presenting to other primary care clinicians such as medical practitioners or physiotherapists could conceivably be different to those presenting to chiropractors.

2) Objectives: Line 65. Unclear of the relevance of the statement “Specifically to identify individuals at higher risk of developing chronic or recurrent spinal pain”. This objective does not appear to have been addressed in the analysis of the feasibility study or the presented data, so suggest re-word or omit.

3) Line 81-82 – sample size justification is too vague. The specific parameters should be provided, or if the sample size was one of convenience then this could be stated.

4) Line 120-124: it would be helpful to report in the results how many questionnaire results were obtained via telephone as opposed to the primary (online) data collection method. This is of interest to overall feasibility and data consistency.

5) Table 2 – multiple abbreviations are used in the table with no definition at the bottom of the table.

6) Conclusions – slow recruitment is the only feasibility concern mentioned, but clearly there are major concerns about a non-representative sample being included in the study (eg. very narrow study setting, only 3-4 chiropractors enrolling most of the participants, inclusion of potentially ineligible participants, no data kept on the number of adolescents who presented, were screened, found to be ineligible or who declined to participate). A larger study would clearly need to improve the representativeness of the sample to be a good use of resources, so this should be mentioned too in the conclusion.

VERSION 1 - AUTHOR RESPONSE

Reviewer: 1

Dr. Noa Ben-Ami, Ariel University

Comments to the Author:

This paper presents an important feasibility study addressing a critical issue, as chronic or recurrent back pain affects approximately 25% of adolescents and has a significant impact on their quality of life.

Background: The background is concise and well-articulated. However, it would benefit from a brief mention of how adolescent back pain may differ in nature, progression, or response to treatment compared to adults.

> Author response: We have added the following to the end of the second paragraph (Line 139, marked copy). "Unlike in adults, the prognosis, clinical course and response to treatment for adolescents with spinal pain may be influenced by developmental factors, such as biological maturity and psychosocial changes.[10, 11] Accordingly, applying findings from the extensive adult research to younger populations would be problematic, highlighting the need to expand the evidence base on spinal pain in adolescents.[12, 13]"

Methods:

Line 76-77: The sentence, "were invited to participate until 20 had consented to recruit adolescents," is unclear. Please consider rephrasing for clarity.

> Author response: We have rephrased the sentence as follows (Line 180, marked copy). "Between May 12, 2021, and December 12, 2022, we invited chiropractors in Sydney, Australia, to participate in the study on an ongoing basis until a total of 20 agreed to recruit adolescents."

Line 80: 45 adolescents overall?

> Author response: Yes, we have revised the original text to the following (Line 183, marked copy).

"We sent fortnightly emails to remind chiropractors of study protocols and to sustain motivation for participant screening. Recruitment continued until the target sample size of 45 adolescents had been achieved."

Line 94: The adolescents got chiropractors' usual clinical care. So, it will be the clinical course of spinal pain in adolescents with chiropractors' usual clinical care. I think it will be good if you have a control group with no treatment to see the usual clinical course of adolescent back pain. for your consideration.

Author response: We agree including a no-treatment control group to observe the natural course of adolescent back pain is a valid approach. However, it falls outside the scope of this feasibility study. Our focus was assessing the feasibility of methods to track the clinical course of spinal pain in a chiropractic setting. We will consider this in the design of our future more extensive cohort study.

Line 174: It is surprising that the sample did not follow the typical gender distribution observed in similar studies, where females often outnumber males. This anomaly warrants further discussion.

Author response: We agree there is substantial evidence to support females more commonly report spinal pain than males, thus being the dominant sex in many published studies. We have revised our discussion under the subheading *Generalisability* to acknowledge this more clearly (Line 405, marked copy).

"Several factors contributed to the non-representativeness of our sample, including recruitment methods (participants recruited from 10 chiropractors in Sydney, Australia), the study population (adolescents with moderate-to-severe spinal pain seeking chiropractic primary care), and study-specific challenges (limited data on potentially eligible participants). For instance, our sample had 43% females, which differs from previous spinal pain research, where females typically predominate.[27]"

Line 212 – Recovery rates appear notably low, with only 31% of adolescents recovering after six weeks. In contrast, the recovery rate in adults is typically around 70%. It will be good for discussion.

Author response: We have revised our original text discussing recovery rates under the subheading *Spinal pain clinical course* in the Discussion (Line 387, marked copy) and compared our findings to similar studies. We have also emphasised that while this is an interesting finding, our 12-week study period does not capture the full clinical course of spinal pain in adolescents.

"One-third of participants recovered from their enrolment episode of spinal pain, of whom 64% experienced another episode before the study concluded. This aligns with Dissing et al.'s findings, where one-quarter of 1,465 8-14-year-old Danes experienced three or more episodes of spinal pain annually over three years [25]. Additionally, Dissing et al. found the more episodes a young person had, the longer each episode lasted. Aartun et al.'s two-year longitudinal study of 1,042 Danish adolescents aged 12-15 similarly found adolescents with higher intensity pain (>4/10 NRS, as in our cohort) experienced more frequent episodes [26]. These findings emphasise the limitation of our 12-week study period in capturing the full clinical course of moderate-to-severe spinal pain in adolescents and identifying individuals at higher risk of chronic or recurrent spinal pain."

The discussion is thorough and provides valuable insights. The limitations are well-recognized, adding transparency and credibility to the study.

Author response: Thank you.

Reviewer: 2

Dr. Andrew Hahne, La Trobe University

Comments to the Author:

This feasibility study is of interest as the population (adolescents with spinal pain) is not widely studied and in fact most clinical trials exclude this population. With some minor revisions the paper could better achieve its purpose of informing the feasibility of future research in this area.

Overall, the high rate of follow-up and retention was promising. Concerns with recruitment rate and sample representativeness are declared in the paper, although strategies to avoid them in a larger study could be more concrete.

Author response: We have added the subheading *Solutions* in our Discussion and set out a paragraph that provides recommendations for future cohort studies (line 376, marked copy).

"To address the problem of low recruitment, we propose expanding clinician involvement beyond chiropractors to include physiotherapists and general practitioners, as well as specialised clinics such as spinal pain or chronic pain centres. By identifying high care-seeking settings before commencement, we could strategically allocate resources to support higher-yield recruitment, i.e. embed research staff in clinics to ensure consecutive and accurate screening, enrolment, and timely collection of baseline data. Also, if we engage with practitioners earlier in planning study procedures, we can better understand and respond to the factors influencing active participation. To improve the accuracy of consecutive screening and data recording for ineligible patients, compensating clinicians for their time spent on screening and data entry should be strongly considered rather than solely for patients' successful enrolment and completion [25]."

In our discussion under *Generalisability*, we also discuss solutions for representativeness (line 409, marked copy).

"In a larger cohort, recruiting an Australian-wide sample from various healthcare settings would likely produce a more representative sample from diverse backgrounds, both rural and metropolitan areas and high and low socioeconomic backgrounds, supporting better generalisability."

Specific suggestions for improving the reporting of this study:

1. The setting of the study (chiropractic care) should be mentioned in the title, objectives and conclusions. This is often labelled in the article too generally as "Australian primary care", of which the chiropractic setting represents only a small proportion. Adolescents presenting to other primary care clinicians such as medical practitioners or physiotherapists could conceivably be different to those presenting to chiropractors.

Author response: We agree and have revised all references to 'chiropractic care' or 'chiropractic setting' throughout the manuscript, including the title, objectives and conclusions.

2) Objectives: Line 65. Unclear of the relevance of the statement "Specifically to identify individuals at higher risk of developing chronic or recurrent spinal pain". This objective does not appear to have been addressed in the analysis of the feasibility study or the presented data, so suggest reword or omit.

Author response: We have omitted this sentence.

3) Line 81-82 – sample size justification is too vague. The specific parameters should be provided, or if the sample size was one of convenience then this could be stated.

Author response: The sample size for this study was determined based on pragmatic considerations including available resources to recruit clinicians and participants and conduct data collection. Additionally, it was chosen to achieve an expected response and retention rates of approximately 85%, informed by prior experience with similar studies. To provide more specific parameters, we estimated that with n = 45, a retention rate of 89% (accounting for n = 5 attrition) would provide sufficient precision within a 95% confidence interval (CI). At this sample size, the lower bound of the CI would remain at approximately 80%, which was considered an acceptable level of precision for this feasibility study. We have revised the manuscript accordingly to include these details (line 185, marked copy).

"The sample size of 45 was pragmatically determined based on available resources (including time, funding and logistical considerations) and prior experience. We estimated an 89% retention rate, accounting for 5 participants expected to drop out, providing sufficient precision within a 95% confidence interval, with the lower bound remaining around 80%. [19, 20]."

4) Line 120-124: it would be helpful to report in the results how many questionnaire results were obtained via telephone as opposed to the primary (online) data collection method. This is of interest to overall feasibility and data consistency.

Author response: Great point. We have included this information in our results as follows (line 273, marked copy).

"Table 1 outlines *response rates* and completion times for each data collection instrument. For Twilio compatibility, the mobile phone numbers for 33 participants (73%) had to be corrected to international format. The average response rate to all weekly SMSs was 94% (89% to 98%). In 1% of all SMS data measures (n=10 of 990 total possible SMSs), adolescents actively returned empty data; these were not treated as non-responses. Phone reminders resulted in completing two full and two partial baseline questionnaires (n = 4 of 45, 9%), 12 weekly SMSs (n = 12 of 990, 1%) and no follow-ups. One quarter (27%) of adolescents received assistance completing their baseline questionnaire, 17% with weekly SMSs, and 14% with their final follow-up questionnaire."

As well as in our discussion (line 347, marked copy).

"While SMS data collection was largely effective, the most significant challenge was ensuring mobile phone numbers were entered in the correct format. This issue could be addressed in future studies by enforcing a restricted entry format rather than relying on instructions alone."

5) Table 2 – multiple abbreviations are used in the table with no definition at the bottom of the table.

Author response: We have added all abbreviations to the legend as follows or have written in full in Table 2.

“Legend: AU$; Australian dollar, cm; centimetres, CT; computed tomography, EOS; low-dose, weight-bearing X-ray technology, GJH; generalized joint hypermobility, mean±SD(range); mean, standard deviation and range for normally distributed data, median(range); median and full range for skewed data, median(IQR); median and interquartile range for skewed data, MRI; magnetic resonance imaging, n(%); number of participants and percentage of sample, NRS; numeric rating scale, PedsQL; Pediatric Quality of Life, SP; spinal pain, US; ultrasound, X-ray; radiograph.”

6) Conclusions – slow recruitment is the only feasibility concern mentioned, but clearly there are major concerns about a non-representative sample being included in the study (eg. very narrow study setting, only 3-4 chiropractors enrolling most of the participants, inclusion of potentially ineligible participants, no data kept on the number of adolescents who presented, were screened, found to be ineligible or who declined to participate). A larger study would clearly need to improve the representativeness of the sample to be a good use of resources, so this should be mentioned too in the conclusion.

Author response: Thank you for outlining these concerns for a future cohort. Per our response to Reviewer 1 above, we have expanded our discussion under the subheading *Generalisability* to acknowledge these points (Line 404, marked copy).

“The preliminary nature of this feasibility study warrants caution regarding generalisability. Several factors contributed to the non-representativeness of our sample, including recruitment methods (participants recruited from 10 chiropractors in Sydney, Australia), the study population (adolescents with moderate-to-severe spinal pain seeking chiropractic care), and study-specific challenges (limited data on potentially eligible participants). For instance, our sample had 43% females, which differs from previous spinal pain research, where females typically predominate.[28] In a larger cohort, recruiting an Australian-wide sample from various healthcare settings would likely produce a more representative sample from diverse backgrounds, both rural and metropolitan areas and high and low socioeconomic backgrounds, supporting better generalisability.”

We have also updated our conclusion (line 433, marked copy).

“Our feasibility study's high response and retention rates indicate the viability of our methods for a large-scale prospective cohort study exploring the clinical course of moderate-to-severe spinal pain in adolescents seeking chiropractic care. Refinements to address low recruitment and sample generalisability will be essential for the success of a larger study.”
